# Meaningful Gesture in Monkeys? Investigating whether Mandrills Create Social Culture

**DOI:** 10.1371/journal.pone.0014610

**Published:** 2011-02-02

**Authors:** Mark E. Laidre

**Affiliations:** Department of Ecology and Evolutionary Biology, and Neuroscience Institute, Princeton University, Princeton, New Jersey, United States of America; Georgia State University, United States of America

## Abstract

**Background:**

Human societies exhibit a rich array of gestures with cultural origins. Often these gestures are found exclusively in local populations, where their meaning has been crafted by a community into a shared convention. In nonhuman primates like African monkeys, little evidence exists for such culturally-conventionalized gestures.

**Methodology/Principal Findings:**

Here I report a striking gesture unique to a single community of mandrills (*Mandrillus sphinx*) among nineteen studied across North America, Africa, and Europe. The gesture was found within a community of 23 mandrills where individuals old and young, female and male covered their eyes with their hands for periods which could exceed 30 min, often while simultaneously raising their elbow prominently into the air. This ‘Eye covering’ gesture has been performed within the community for a decade, enduring deaths, removals, and births, and it persists into the present. Differential responses to Eye covering versus controls suggested that the gesture might have a locally-respected meaning, potentially functioning over a distance to inhibit interruptions as a ‘do not disturb’ sign operates.

**Conclusions/Significance:**

The creation of this gesture by monkeys suggests that the ability to cultivate shared meanings using novel manual acts may be distributed more broadly beyond the human species. Although logistically difficult with primates, the translocation of gesturers between communities remains critical to experimentally establishing the possible cultural origin and transmission of nonhuman gestures.

## Introduction

Around the world, human populations exhibit dramatic differences in behavior, much of it independent of ecology and genetics [1], [2]. The concept of culture was originally formulated to describe such geographic differences in the behavior of human communities [3], essentially variation in “the way we do things” [4], [5]. Recently, however, the culture concept has been applied to examine parallel cases discovered in nonhumans [6]–[12]. Despite substantial wrangling over definitions of ‘animal culture’ as well as disagreement over whether animals should even be accorded culture [6] many scientists agree that, at least in some nonhuman species, individuals may possess a capacity to learn from others within their community. This capacity for social learning in animals can potentially generate population-level phenomena that bear some similarities to patterns of human culture [11]. One possible population-level consequence of social learning is that, over time, the behavior of an animal community can diverge from that which is found in other communities of the same species, resulting in a prominent between-community differences and within-community similarities in patterns of behavior [11]. Animal culture, in this minimalist sense, thus exists when socially-learned behavior that is shared in one animal community is absent in other communities of the same species [10], [12].

Biologists, psychologists, and anthropologists have now begun to systematically investigate the underlying processes and the ultimate products of culture across a diversity of species, comparing and contrasting mechanistic and functional elements of both animal and human culture to uncover how and why cultural abilities evolved [9], [13]. Perhaps the most compelling cases of animal culture that have been identified thus far are those that center on how social interactions are mediated within a community. Unlike cases of animal culture in the foraging or technological domain (which can sometimes be readily explained as mere corollaries of local habitat) cases of animal ‘social culture’ appear to have less grounding in pure ecology [14]–[16]. A community, for instance, may conduct its affairs in a certain way while other communities never employ this way or use different ways, irrespective of environment [17]. Our closest evolutionary relatives, the nonhuman primates, have provided some of the strongest evidence for social culture [15]. A paradigm example is the so-called ‘grooming hand-clasp’ of chimpanzees [18]. This unique behavior, in which two apes clasp their hands overhead during mutual grooming, has been regarded as the “the first serious claim of culture in animals” [Ref 9, p.341]. Handclasp grooming has been observed extensively within some chimpanzee communities, both captive (e.g., Yerkes Regional Primate Research Center in Georgia) and wild (e.g., Taї Forest, Ivory Coast; Mahale, Tanzania; Kibale Forest, Uganda); but the behavior has never been detected elsewhere despite long-term observation of other communities with similar environments and genetic compositions (e.g., Bossou, Guinea; Gombe, Tanzania; Budongo Forest, Uganda) [18]–[20]. Notably, different chimpanzee communities that exhibit handclasp grooming have also been found to vary in the fine-grained nuances of how they perform the gesture, suggesting some degree of cultural standardization in ‘style’ within each local community [21].

In our own species, variable modes of carrying out social interactions between different communities are often imbued with a deeper significance; and it is this added layer of meaning that makes these acts truly cultural, shared collectively by a community as whole [22]. One class of behaviors that can possess such meaning and that are found in human and nonhuman alike are ‘gestures,’ behaviors which Smuts [Ref 23, p. 301] defines as broadly encompassing “all nonvocal actions with potential communicative significance.” Some gestures, for instance manual gestures, involve movements of the hands and arms in the vicinity of conspecifics, possibly exerting social or communicative effects. For a gesture, manual or otherwise, to qualify as ‘meaningful’ though it should be demonstrated to influence others' behavior, altering their response pattern by either conveying information about the gesturer's mood or otherwise manipulating what onlookers do [24], [25]. While it has been proposed that handclasp grooming might be a meaningful gesture, symbolizing close dyadic relationships [16], [26], to date no systematic analysis has been carried out to investigate what, if any, meaning this gesture holds for chimpanzees. Moreover, although other cases of cultural gestures in nonhuman primates have been discovered in addition to handclasp grooming [27], these cases have involved mostly similar kinds of tactile gestures that operate intimately at close-range, such as scratching [28], grooming [29], or sucking and sniffing of others' body parts [30], behaviors which it is less than clear should qualify as ‘meaningful’. Few cultural gestures thus seem to exist in nonhuman primates that operate visually from a distance to impart a meaning that is shared by a local community as a whole [20], [31].

The most likely candidate taxon for possession of such a gesture would seem to be apes, since sparse evidence has been available for any type of manual gesturing in monkeys [16], [32]–[34], let alone the creation of gestures de novo [35]. Notably, ape gesturing has been studied in detail across several populations of all four species of great ape [33] and these studies have revealed complex patterns, including sensitivity to the audience's attentional state and comprehension [36], [37], referential communication about external objects in the environment [38], and cultural variation in the presence of certain gestures across groups [27]. Given how few intensive studies have investigated possible monkey gestures across different groups of the same species, it is perhaps not surprising that, outside of humans, nearly all culturally-based gestures identified thus far have been restricted to apes [16]. It has been maintained, therefore, that: “free hand gestures are a unique feature of ape and human communication; they are not found in the monkeys” [Ref 27, p.19.] Recently, however, new cases of relatively sophisticated monkey gesturing [30], [39] have been revealed, raising the possibility that it is within monkeys' reach to culturally-craft a gesture for meaningful communication over a distance.

The present paper investigates a community of mandrills' (*Mandrillus sphinx*) unique gesture, hitherto unreported, which has persisted stably for a decade within a single community and has never been observed in any other communities of the species across three continents. In this paper I examine (1) whether the gesture is meaningful, in the sense of possessing a communicative function that involves socially influencing other community members from afar and (2) whether the gesture might qualify as a form of animal social culture. In addressing these two questions I (a) detail the form and temporal dynamics of the gesture; (b) catalogue the individuals that make use of the gesture; (c) provide the available history of the gesture's emergence and spread; (d) describe the circumstances surrounding one of the gesturer's deaths and a modification to the gesture that users subsequent-to-the-originator have added; (e) isolate the contexts in which the gesture occurs and the responses it elicits in others; and finally (f) I suggest new experiments that might shed light on how cultural forces may shape the way nonhumans perceive and propagate gestures.

## Results and Discussion

**Figure 1:**
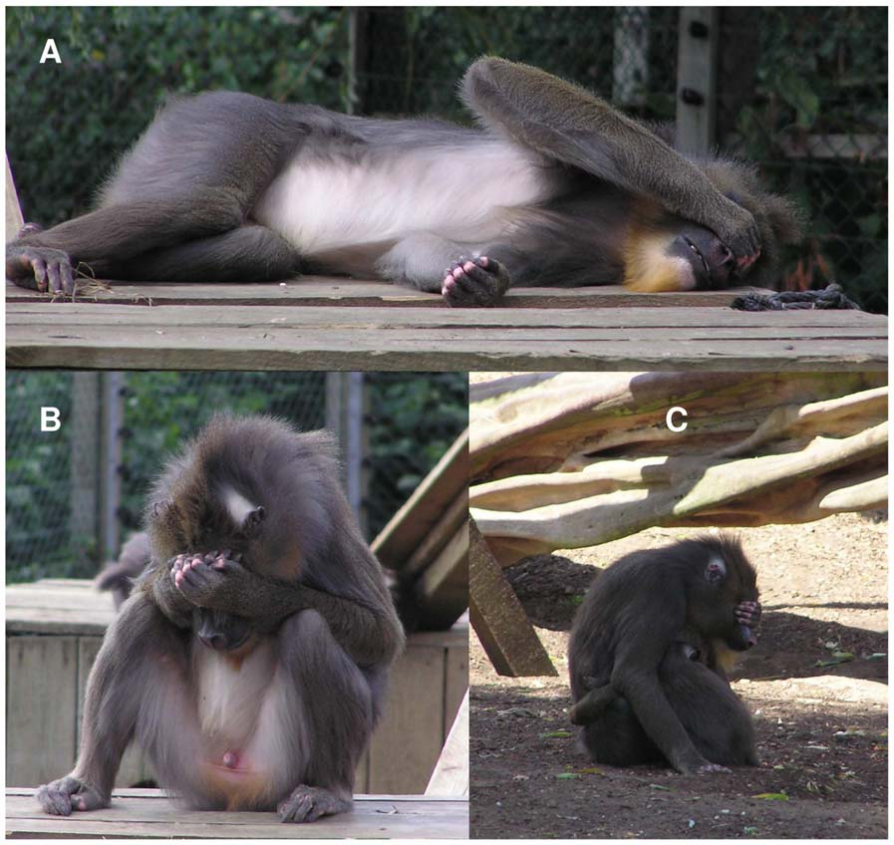
The unique Eye covering gesture of the Colchester community. (**A**) A male performs the gesture with his right hand while lying and while lifting his elbow prominently as a ‘flag’. (**B**) Another male performs the gesture while sitting with both hands held over his eyes, the left hand on top. (**C**) A female (the originator) performs the gesture with her left hand while sitting embraced with one of her offspring in the shade.

Mandrills, the largest of all monkeys, are endemic to the rain forests of equatorial West Africa and are housed in captive groups around the world. In a captive community of 23 mandrills in Colchester, England seven individuals (an adult female and six males of various ages; Table 1, S1, S2) were observed performing a prominent gesture in which one or both hands were brought overtop the face, covering the eyes (Figure 1; Video S1, S2, S3). This ‘Eye covering’ gesture was unique, not being observed in any of eighteen other mandrill groups distributed across the USA, Gabon, the United Kingdom, Germany, and Italy (Table S3). In addition to these groups, observed by the author over the course of 9 years, other colleagues commented on further groups (personal communications in Table S3), which were found in Israel, Gabon, Italy, the Czech Republic, and Belgium, some of which had been observed over multiple generations for more than a decade. Nevertheless, the Eye covering gesture has never been observed outside of Colchester, and nor has it been reported in any of the publications of prior mandrill researchers, despite multiple independent investigations of yet other communities of this species (Table S3).

**Table 1:** Dominance ranking of individuals in the Colchester community ^A^.

Dominance ranking	*Name* (sex, age at study start, birth date)
Highest	*Dume* (♂, 12 yrs, born 15 Sep 1994 at Southport Zoo and transferred in 23 Jan 2004) *Alpha male
	*Celine* (♀, 22 yrs, born 3 Jan 1985 at Zoological Society of London and transferred in 27 Oct 1988) *Died 22 May 2009
	*Orinoko* (♀, 13 yrs, born 8 Nov 1993)
	*Oakley* (♀, 6 yrs, born 4 Feb 2001)
	*Malaya* (♀, 23 yrs, born 14 Aug 1984 at Paignton Zoo Environmental Park and transferred in 27 Oct 1988)
	*Matilde* (♀, 6 yrs, born 21 Sep 2000)
	***Milly (♀, 10 yrs, born 27 Sep 1996) *Originator and most prolific gesturer
	*Solomina* (♀, 14 yrs, born 27 Sep 1992)
	***Phoenix (♂, 10 yrs, born 12 Oct 1996) *Died 26 Jun 2008
	***Mac (♂, 9 yrs, born 18 May 1998)
	***Max (♂, 9 yrs, born 10 Jul 1998)
	***Barney (♂, 7 yrs, born 8 Oct 1999)
Lowest	***T.J. (♂, 5 yrs, born 22 Sep 2001)

A Only individuals ≥3 yrs of age at the start of the study are listed. All listed individuals were born at the Colchester Zoo unless noted otherwise. The names of gesturers (those who performed Eye covering) are written in bold with three asterisks next to them. The gesturer ‘Kayin’ (♂, 1 yr old at start of study, born 13 Feb 2006) is not shown, since his dominance rank was changing throughout the course of the study.

In the Colchester community, the Eye covering gesture was first observed in 1999 when a female ‘Milly’, then 3 yrs old, began performing the gesture spontaneously (personal communications from Liz Butcher, keeper during 1995–2000; Kirsty Stewart, keeper during 2000–2006; and Kate Harness, keeper during 2006-present). Neither during the gesture's origin nor anytime since has there been any human intervention to elicit or in any way encourage the mandrills to gesture (ibid; and personal communications from Sarah Forsyth, Curator, and Rebecca Perry, Director of Conservation, Education and Research at the Colchester Zoo). Mandrills perform Eye covering irrespective of whether humans are in the viewing vicinity of their exhibit, and the gesture has never once been observed being deployed in any human-geared contexts, as while being fed. Presently, the gesture's originator Milly, who is now an adult, continues to perform Eye covering regularly, as do several younger individuals within the group who began performing the gesture after Milly but are not her offspring (see Table S1 for timeline of acquisition). During two study periods separated by 1 yr I quantified how this unique gesture was used and examined its potential communicative value within the Colchester community.

Eye covering was performed frequently (Mean ± SE: 6.3±4.4 times per h in focal samples across all community members ≥3 yrs of age; N = 13 individuals; Table 1 and S1). Up to three individuals at a time were observed performing the gesture concurrently, each at their own separate location and not apparently in any relation to one another. Unlike relatively instantaneous behavior, such as scratching, the Eye covering gesture was held continuously for extended periods, lasting uninterrupted up to 17 min and 6 s, or more than twice as long as the longest reported handclasp grooming bout in chimpanzees [19], [26]. The exact time between onset and offset of the gesture averaged 56±7 s (N = 275 from focal sampling). Typically though several Eye covering bouts were performed one after the other with only brief separations in which the gesturer switched hands or temporarily removed and replaced its hand while changing position. When such adjacent instances of gesturing (<1 min separation) were combined (henceforth termed ‘lumped bouts’), the duration of gesturing was substantial, averaging 8.8±1.5 min (range: 2 s – 36.2 min, N = 33; see Table S1 for data on each gesturer separately).

Holding the hand in place for so long might not entail negligible effort. Indeed, potential effort was also exhibited in another aspect of the gesture: individuals that were gesturing sometimes simultaneously elevated their elbow high in the air, keeping it aloft like a ‘flag’ (Figure 1A; Video S1). Besides the originator, who has never been observed lifting her elbow during the gesture, five of the six other gesturers exhibited this conspicuous behavior (Table S1), executing it exclusively during Eye covering. Elbow raising occurred in 31.5% of N = 89 lumped bouts that were performed by gesturers other than the originator, and the raised elbow was held in place on average 1.0±0.2 min (range: 0.03 – 3.4 min; N = 42 elbow raises from focal sampling). The possible muscular exertion involved in Eye covering and Elbow raising suggested a plausible function, one that might favor notifying others and amplifying the detectability of the gesture via an embellishment. Consistent with such social signaling, the gesture's function could not be reduced to basic environmental factors, like blocking light from entering the eyes: only 34.6% (of N = 208 gestures) were performed while the gesturer was in direct sunlight; all the rest were performed in the shade cast by opaque building structures or overhanging branches (39.9%), or when the sky was completely overcast (25.5%). In contrast to the lack of evidence for a sun-shielding function, observational evidence suggested that Eye covering might have a significant social function, mediating interaction among conspecifics.

### Contextual and Response Patterns

**Figure 2:**
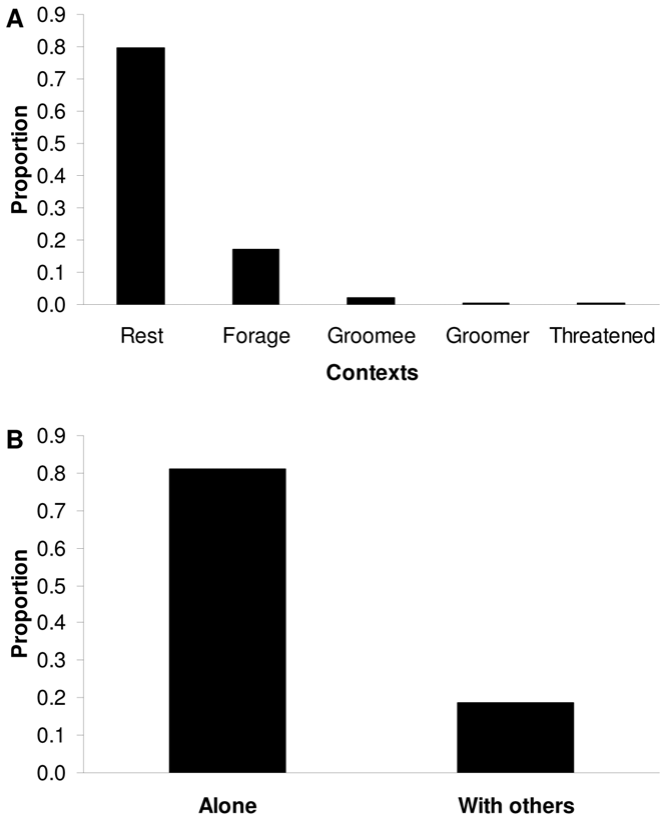
Contexts surrounding the gesture's use. (**A**) Behavioral contexts (see Materials and Methods) in which the gesture was performed at least once. Based on N = 359 gestural occurrences from focal and behavioral sampling combined. (**B**) Whether the gesturer was alone or had other community members within 2 m at the time its gesture was first detected. Based on N = 266 occurrences from behavioral sampling. Included in the ‘alone’ category are instances in which an adult female was gesturing and was alone other than having her fully-dependent infant, which always remained embraced with or in arm's reach of her.

The social function of Eye covering did not appear to be geared toward initiating interactions: the gesture was never performed while individuals were locomoting or while they were standing, poised for interaction. Rather individuals were always stationary while gesturing, either lying (27.6%; Figure 1A) or sitting (72.4%, N = 359; Figure 1B, 1C). Interestingly, whereas most nonhuman primate gestures identified thus far, including non-cultural ones, have been embedded in intensely social activities, like agonism, play, or intimate bonding [32]–[34], the Eye covering gesture occurred primarily during rest, defined by the absence of any socializing between the gesturer and others (Figure 2A). Rest, however, did not imply actual sleep, and it was clear from several factors that gesturers did not necessarily keep their eyes closed while covering them. For instance, gesturers would frequently glance back and forth, apparently peeking through cracks between their fingers to survey the locations of other community members. And when a more dominant group mate approached, a gesturer would orient its gaze toward that dominant, immediately taking its hand off and avoiding if the dominant came too close. Gesturers also sometimes held their hands slightly away from their face (Video S2) or their hands sometimes gradually slid down their muzzle during the course of an extended gesturing bout, in both cases revealing open eyes. Thus, despite engaging in hardly any social activities while performing the gesture, gesturers nevertheless remained visually aware of their surroundings.

**Figure 3:**
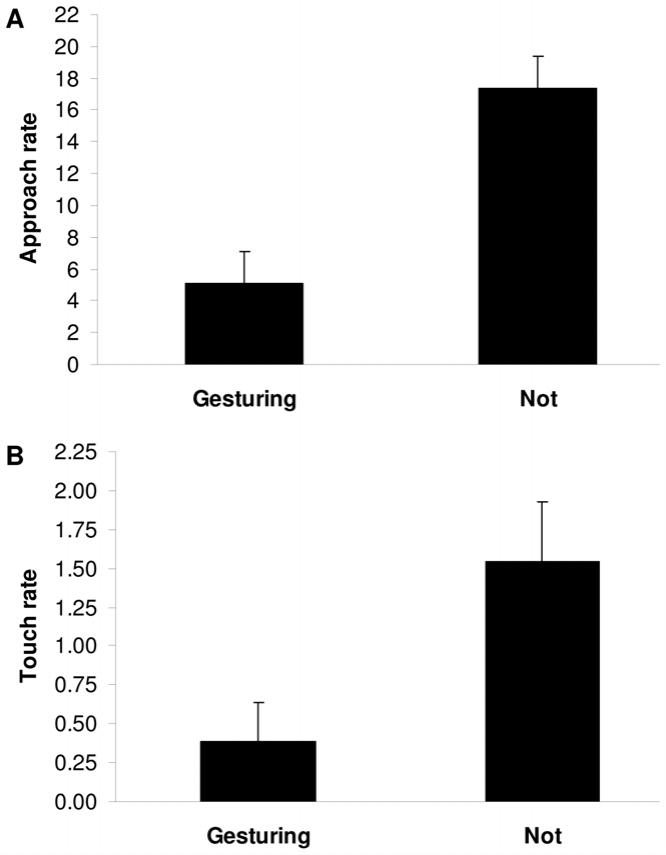
Individuals experience significantly reduced disturbance while gesturing. Disturbance rates (per h) when mandrills were and were not gesturing. Periods of non-gesturing represented controls in which the same individuals were in a stationary resting context with either a lying or sitting body posture, but were not covering their eyes. (**A**) Approach rate (others coming within 2 m of the focal individual). (**B**) Touch rate (others initiating tactile contact with the focal individual). Mean + SEM shown. See Tables S4 and S5 for data on each individual separately.

The sustained vigilance of gesturers in the absence of social involvement raises the possibility that Eye covering might serve to reduce the amount of disturbance a gesturer received. Such a function could be useful during periods when engagements with others are undesirable. To examine this possible anti-social, disturbance-reducing function I collected data both by behavioral sampling [40] (in which the presence or absence of other community members was quantified in the vicinity of observed gestures) and by focal animal sampling [41] (in which instances of received approaches and touches were recorded continuously while the same individuals were and were not gesturing). Results showed that if an individual was gesturing, then in most cases it was free of the company of other community members (Figure 2B). Furthermore, gesturing appeared to inhibit others from disturbing the gesturer: individuals received significantly fewer approaches (*t*-test assuming unequal variances: *t* = 4.30, *df* = 6.89, *p* = 0.0037; Figure 3A) and significantly fewer touches (*t*-test assuming unequal variances: *t* = 2.49, *df* = 6.53, *p* = 0.0440; Figure 3B) from other community members when they were performing the gesture compared to control periods when they were not performing the gesture but were otherwise postured similarly. Since the gesture was performed throughout the Colchester mandrills' entire habitat, these effects were not simply a result of individuals positioning themselves in some isolated locale while they gestured. Indeed, the gesture was performed most commonly in the center area of the enclosure, where the majority of the community and hence the majority of potential disturbances tended to be (Laidre, personal observation).

By covering their eyes with their hands, individuals possibly conveyed to others that they wanted to be left alone, and this message may have been respected as a ‘do not disturb’ sign. Regardless of a gesturer's rank, its hands were never removed from its eyes by another individual. It is notable though that the individuals who employed the gesture tended to occupy the bottom of the dominance hierarchy (Table 1), and thus would have benefited from regulating social disturbances. In particular, the five older male gesturers occupied the lowest five positions among the thirteen mature members of the Colchester community. Given these males' subordinate rank, approaches and tactile interactions could preface costly attacks and persecution from others, especially from higher ranking individuals. Indeed, within the period of the study, one of these males, Phoenix, was so severely wounded in an attack by a superior that he had to be euthanized (Table 1, S1). Similarly, the originator Milly was the second lowest-ranking among seven adult females. The pressure for this particular female to reduce social disturbances may have been especially strong at the time of the study, since she had a dependent infant as well as another older offspring, and would have benefited from moderating how often others interfered with her vulnerable progeny. Notably, many of this female's gestural performances (41.3% of N = 138 from behavioral sampling) were performed while she was embraced with or in arm's reach of one of her offspring but was otherwise separated from third parties. In these cases the gesture may have functioned to prevent third parties from interrupting her dyadic kin interactions. Finally, the seventh gesturer was an immature male who only began performing the gesture in the second study period. Given this male's young age and high social activity levels, it was unclear what utility the gesture could have for him. He executed the gesture on only five occasions, but on each occasion specifically after positioning himself within a meter of one of the older male gesturers who, at the time, was currently performing Eye covering or had performed it just moments before.

### Reasons for Uniqueness

Irrespective of whether the Eye covering gesture is meaningful or not, its uniqueness to the Colchester mandrills raises the question of why all the performers of this gesture should be restricted to just a single community. None of several hundred other mandrills (Table S3), including ones from communities with virtually identical social compositions to Colchester, ever covered their eyes, not even for a few seconds. And Eye covering has also never been observed among wild mandrills [42; Kate Abernethy, personal communication]. The absence of Eye covering in these other communities does not appear explicable based on insufficient sampling, human inducement, genetic variation, or ecological differences across communities. Sampling error, for instance, is an unlikely explanation for the gesture's absence at every other site, since some communities were observed over extended periods lasting across generations. Human inducement likewise is unlikely: despite the folk adage ‘monkey see, monkey do’ there has so far been no successful attempt in any study to train monkeys on the ‘do-as-I-do’ paradigm [43], in which animals are rewarded for copying the actions of a human. Monkeys, unlike great apes, dolphins, and dogs [43]–[48], thus evidently will not mimic human gestures they might happen to see. Also, neither genetic or habitat differences between communities appear to be viable explanations for the gesture's exclusive confinement to Colchester. In terms of genetics, gesturers had diverse parentage (Table S2), with as few as three generations connecting the Colchester community back to a wild gene pool; and captive management practices have ensured that breeding between different zoo mandrill communities occurs regularly, so hereditary differences do not accumulate. Likewise, habitat features and living conditions between the Colchester community and many of the other communities were broadly similar in design, having been arranged for the very same purpose of providing a safe environment for the animals where humans could easily watch them. The only environmental parameter that might be deemed relevant—density—was not higher in Colchester, which was the eleventh most dense of nineteen total study groups (Table S3). Accordingly, Eye covering did not emerge merely through convergent asocial responses to an elevated density. Overall, therefore, the most plausible explanation for the gesture's confinement to the Colchester community is that the gesture was, at least in part, culturally transmitted after its innovation by the originator Milly. The usage of such a peculiar manual act exclusively by seven individuals belonging to the same community makes it improbable that each of these seven separately fabricated the behavior. Abundant opportunities would clearly have been available for novices to notice and be influenced to reproduce the gesture, given its frequency and lengthy bouts.

As yet though, there is no irrefutable experimental evidence for cultural transmission of the Eye covering gesture, let alone for any gesture in a nonhuman [33]. To date, only one experiment has been carried out to critically examine how nonhumans might socially learn their gestures from conspecifics. This study [49] trained two chimpanzee subjects to demonstrate an unusual gesture for requesting food from a human, finding no evidence that other chimpanzees ever acquired the gesture. The experiment is limited, however, in two key respects. First, the focal gesture had essentially no naturalistic relevance to the lifestyles of the chimpanzees, as it was not something they themselves came up with for intra-specific interactions but rather something humans trained them to execute arbitrarily for inter-specific interactions. Second, recent research has suggested that social learning in nonhuman primates is grounded in an inherent motivation to copy others that is independent of extrinsic rewards, like getting food, perhaps stemming from a desire simply ‘to belong’ and ‘fit in’ within one's social community [11], [16], [50]. Gestures whose purpose lies in obtaining rewards from a human would seem ill-suited to inducing such a social motivation to be like others. Clearly, more experiments are needed with less artificial—but still equally unique—gestures, ones like Eye covering that seem endowed with social relevance.

Ultimately, it may be possible to employ experimental approaches with Eye covering, complementing and extending the ‘method of exclusion’ [51] of the present paper. For instance, future inter-zoo animal transfers, in which gesturers are translocated to new communities, could be opportunistically exploited as ideal tests of the sort that have rarely been feasible with primates [10], [52], [53]. Compelling evidence for social learning and cultural dissemination could then be gleaned if new communities which previously lacked the gesture later began performing it following the arrival of the transplanted gesturer. Equally informative could be experiments inside the Colchester community itself. Video playback, for instance, could be used to determine what responses Eye covering elicits compared to other gestures. And removal experiments as well as the provision of visual barriers could be used to test the flexibility of gesturers, determining whether they cease the Eye covering gesture if they are not liable to be disturbed or if others would be incapable of seeing their gesture. Finally, of foundational importance is continued, long-term monitoring within the Colchester community itself. For while acknowledging that non-experimental methods of characterizing animal culture are limited [8], [54], it is only through such longitudinal observations that we can document how long animal cultures like Eye covering endure and also detail what, if any, further diffusion dynamics they exhibit as new members become integrated into the community and others are removed or die out.

### Conclusion

For any given primate species, hard-wired facial expressions and body movements can be found across essentially every community in virtually identical form (see [55]–[57] for examples in mandrills). Eye covering, in contrast, is a unique gesture that is characteristic of a single mandrill community in Colchester, England. The gesture occurred several hundred times during the course of the present study, but it has never been seen outside of the Colchester community despite extensive study of other communities, spanning multiple years, observers, and continents. The gesture emerged naturally, independent of human involvement, and has now lasted a full generation, still being performed daily as of Sept 2009 despite demographic changes within the community. That the gesture is shared by a sizable contingent of the community, each of varying age and sex, makes it difficult to account for as a product of several completely independent inventions (the possibility of independent invention has, in contrast, undermined other supposed cases of gestural ‘culture’ or ‘tradition,’ in which the number of performers was fewer [33], [39]). Statistical tests could be useful in showing the improbability of asocial learning [58], [59], though in Eye covering's case this gesture's absence in so many geographically diverse mandrill communities outside Colchester shows convincingly that covering one's eyes is not something mandrills often try. It is plausible, therefore, that the performance of Eye covering by the originator within Colchester served as a model for other community members who later adopted the gesture. Eye covering thus provides an intriguing case of a monkey gesture that may have originated culturally and ultimately may have become co-opted for a signaling function, influencing others' behavior from afar. Continued observation of the Colchester community coupled with video playback and opportunistic translocation experiments can complement the analyses provided herein, delving more deeply into how the gesture's meaning [24] has been constructed and how the gesture itself is propagated. More broadly, increased attention to the gestures of other monkey species may reveal that their gestural abilities have been underestimated relative to that of apes [27] and humans [22].

## Materials and Methods

### Study Period

During two study periods spanning 2007–2008 I observed the mandrills at the Colchester Zoo in the United Kingdom for 100 h, 37 h during Aug 2007 and 63 h during Jul and Aug 2008. Permission for the study was granted by the zoo and observations were carried out between 0930 and 1830 and involved 49 h of focal animal sampling and 51 h of behavioral sampling (see below). In each of the two study periods the gesture was detected within just a few hours from the start of observation. I observed for over 1000 h between Jan 2002 and May 2010 across eighteen other mandrill communities where the gesture was never detected (Table S3).

### Community Composition

At the start of the first study period the community comprised 23 individuals (10 males, 13 females). This consisted of the alpha male (12 yrs in age) who was transferred into the community in 2004; five younger non-alpha males (ranging from 5 to 10 yrs in age); seven mature breeding-age females (ranging from 6 to 23 yrs in age); and 10 immatures (four of which were male and six female, all <3 yrs in age). Dominance among these individuals was established based on approach-avoid interactions. Exact birthdates and ages at the start of the study are provided for all gesturers and for all non-gesturing individuals ≥3 yrs age in Table 1. Pedigree reports detailing the genealogy of each community member as far back as documented by zoo records are also available upon request from the author or by contacting the zoo's research director (email: Rebecca.Perry@colchester-zoo.co.uk).

Between the first and second study period the size of the Colchester community was reduced slightly from 23 to 21 individuals due to a combination of deaths, removals, and births: one male gesturer died (Table 1, S1), four immature females (all non-gesturers) were transferred to a different community (Chester Zoo) for breeding purposes, and one new female and two new males were born into the community (one of the new males being the son of the originator Milly). Since the end of the second study period the most dominant female (a non-gesturer) died (Table 1), a new female was born (18 Jun 2009), and three females (one of them the originator Milly) are currently pregnant (as of Sept 2009). Gesturing has continued in spite of these changes.

### Habitat and Husbandry

In both study periods, and for nearly every existing community member's lifetime, the mandrills have been housed in a large outdoor exhibit-enclosure (30×28 m). The enclosure has several climbing structures (including a tower and ‘clubhouse’ shelters connected by rafters and ropes), as well as natural grass and plants. In the evenings, the mandrills are free to move between this enclosure and their off-exhibit enclosure, which composes two areas (each 15×5 m) that have indoor accommodation and that are directly connected by a gate to the exhibit enclosure. Feeding occurs twice a day at approximately 1000 and 1500, generally with bread or monkey pellets and various fruits and vegetables. The fence (3 m high) that surrounds the mandrills' entire enclosure is electrified and humans are not allowed to enter the enclosure when the mandrills are inside it. Feeding is carried out by first shifting the mandrills to their off-exhibit enclosure while the food is scattered around the exhibit enclosure, after which the mandrills are re-released. Feeding by zoo visitors is strictly prohibited, and water is available ad lib through drinking faucets.

### Contextual Definitions

The definitions of behavioral contexts in which Eye covering occurred were as follows: ‘Rest’ involved an absence of any socializing on the part of the focal individual, though it did not necessarily involve sleep. If sleep is delineated, minimally, by a prolonged closing of the eyes, then in most cases it was impossible to determine if a focal that was gesturing was also sleeping: the eyes were, by definition, occluded from view. There were indications, however, (see main text) that individuals often did not close their eyes while they gestured. ‘Forage’ was identical to ‘Rest’ in that the focal was not socially engaged, except now the focal intermittently picked through grass and other ground material, finding and consuming food items that were naturally available within the enclosure. In ‘Groomee’ the focal was being groomed by another community member, and in ‘Groomer’ the focal groomed another community member (see [55] for specifics on the actions comprising such allogrooming). Finally in ‘Threatened’ the focal was chased or had a threat display [56] directed at it moments before it gestured. I did not observe the Eye covering gesture in any additional contexts besides those just inventoried, all found in Figure 2. See [55] for information on further contexts (like sexual and play interactions), which Eye covering has not been seen in.

### Data Collection and Sampling Regimes

I collected data with a microcassette recorder, noting the exact time (to the nearest second) of each gestural onset and offset, as well as each related behavioral occurrence and context. I then transcribed these voice recordings into an Excel database for subsequent analysis (see below). Data were collected both by focal animal sampling [41] of gesturers and non-gesturers, and by behavioral sampling [40] of the gesture itself. Focal samples were carried out across every member of the community ≥3 yrs of age (N = 13; Table 1, S1), except one individual, the gesturer Phoenix, who died before focal sampling could be carried out on him. Focal sessions were randomized and lasted 10 min each. However, if at the end of a 10 min sample a focal was still in the midst of performing the gesture, then recording continued (up to 1 h), so that the fine-grained temporal dynamics of the gesture would not be missed. Approximately 3–5 h of focal observation were collected per individual.

During focal sampling I continuously followed the focal's behavior, recording the exact times that the focal spent stationary (lying or sitting) vs. moving (encompassing walking and any other locomotion that was interspersed with brief periods of standing). The following recording rule was applied to accommodate ephemeral changes between these two behavioral states: if a focal switched to lying or sitting (from previously moving) for <1 min, then it was deemed a continuation of the original (moving) context. In addition to recording these movement aspects, I also recorded all approaches to the gesturer (defined by a 2 m threshold) and all touches directed toward the gesturer. Touches including both non-aggressive tactile interactions (e.g., brief hand touches or brushes up against the focal's body while passing by) and aggressive tactile interactions (e.g., pouncing off the focal's back, grabbing, hitting, biting, or swiping the focal). Extended grooming bouts, which could involve myriad minute hand picks through a focal's fur, were counted as just single instances of tactile contact. And approaches and touches to mothers from their fully-dependent infants (who needed to nurse and reestablish contact almost constantly) were excluded.

The above information was recorded exclusively during focal sampling. The following additional behavioral parameters were recorded during both focal and behavioral sampling. When an individual gestured I noted: (a) its bodily posture (lying down or sitting up); (b) the behavioral context (definitions above); (c) the hand position of the gesture (left, right, or both hands, and if both hands, then whether one hand was over each eye or which hand was on top of the other hand); (d) whether the elbow was raised in the air (and exactly how long this lasted, if it was during focal sampling); and (e) whether the gesturer was in the shade or (if exposed to the sky) whether it was currently overcast or there was direct sunlight penetrating. Parameters (d) and (e) were only systematically recorded during the second study period.

During behavioral sampling I targeted the gesture, continuously scanning the entire community for any instances of Eye covering. The prominence of the gesture and length of its bouts guaranteed reliable detection during such scans. Unlike in focal sampling, in behavioral sampling I did not note approaches or touches received by gesturers. Instead, upon first detecting each gesture, I recorded whether the gesturer was operationally all alone (i.e., no other community members were within 2 m) or whether there were others within 2 m (excluding a mother's own fully-dependent infant). The exact onset and offset of the gesture was not recorded in behavioral sampling, and so a set of strict criteria were used to differentiate separate instances of Eye covering. When a gesture was observed consecutively by the same individual during behavioral sampling it was only counted as a new instance of gesturing if the gesturer had either: (i) moved to a different location (>2 m away); (ii) changed between lying and sitting; (iii) switched hands (it did not count as a new gesture if the gesturer used the same hand again after having taken it off); or (iv) if there was a change in the context (e.g., from ‘Rest’ to ‘Threatened’).

### Disturbance Rate Analyses

To analyze the gesture's effect on disturbance rates I divided up all the focal time for each individual into three mutually-exclusive periods in which either: (1) the individual was gesturing (this always occurred while gesturers were stationary, either sitting or lying); (2) the individual was not gesturing but was still stationary, either sitting or lying; or (3) the individual was not gesturing and was moving rather than stationary. I then allocated all approaches and touches to each respective time period in which they fell, calculating approach and touch rates for all three categories on each separate individual. The statistical tests for disturbance rate were conservatively conducted at the level of the individual: each member of the Colchester community that had been focal sampled and had performed the gesture provided a distinct data point for the analyses. Figure 3 is based on a comparison of time periods (1) and (2) for the same individuals (Milly, Mac, Max, Barney, and TJ), all of whom were established gesturers, having been gesturing since the time of the first study period or before (Table S1). The gesturer Phoenix, who died in between the two study periods, could not be included since no focal data was garnered on him before he passed. And the gesturer Kayin, an immature, was not focal sampled because he was never known to perform the gesture until the end of the second study period, when he first did so in behavioral sampling. Tables S4 and S5 provide the data on approach and touch rates, respectively, on which Figures 3A and 3B are based. The data in these Tables are presented for each mutually-exclusive time period and for each separate focal individual (both gesturers and non-gesturers). Statistical tests were carried out on JMP® version 8.0 © 2008 SAS Institute Inc., with the alpha level set at 0.05 and each test two-tailed.

### Miscellaneous Notes

When I first encountered Eye covering at the Colchester Zoo during 2007, the mandrills' caretakers and zoo staff were already well aware of its existence. However, they had no reason to think it special and had assumed that mandrills elsewhere spontaneously and habitually perform Eye covering too. It was emphatically affirmed that the Colchester mandrills have never had any direct human interactions where Eye covering or any other gestures were trained or molded whatsoever [see personal communications above in Results and Discussion]. Also, the sole public viewing area where visitors to the zoo are able to watch the mandrills is situated above the habitat, not close-up to or level with the animals; and during my observations the mandrills paid little attention to the visitors. Finally, it is worth noting that an ocular exam carried out on the originator Milly by the zoo veterinarian in spring 2009 revealed no anomalies (cataracts, etc.) that would make her prone to cover her eyes for purely medical, non-social ends (Dr. J. Lewis, personal communication).

## Supporting Information

Table S1 In the Colchester mandrill community, the Eye covering gesture is performed frequently by five members (one now deceased) and occasionally by two additional members. Information about the gesturers is provided below, along with each individual's respective sample size of gestures and other information about their gesturing.
(0.04 MB DOC)

Table S2 Parentage of Colchester mandrills that have performed and not performed the Eye covering gesture.
(0.04 MB DOC)

Table S3 Other study groups outside of Colchester [updated from Table 1 of Laidre 2008, 2009]. Groups are listed chronologically in the order in which they were first observed. Personal communications from other mandrill observers follow at the bottom of the Table.
(0.06 MB DOC)

Table S4 Rate (per h) of approaches received while individuals were gesturing and not gesturing. Times while individuals were not gesturing are divided into stationary periods (focal was sitting or lying) and moving periods (focal was locomoting about).
(0.04 MB DOC)

Table S5 Rate (per h) of touches received while individuals were gesturing and not gesturing. See Table S4 for further explanation.
(0.04 MB DOC)

Video S1 A male gesturing. A male lying and performing the Eye covering gesture during a resting context using his right hand and with his elbow raised.
(5.09 MB WMV)

Video S2 A female gesturing. A female (the originator) sitting and performing the Eye covering gesture during a foraging context using her left hand.
(11.69 MB WMV)

Video S3 A female is disturbed while gesturing. A female (the originator) sitting, embraced with her offspring, and performing the Eye covering gesture using her left hand. She immediately moves away when a third party approaches and attempts tactile interaction.
(14.05 MB WMV)
