# Supplementary material for: Meaningful Gesture in Monkeys? Investigating whether Mandrills Create Social Culture
Source: PLoS One. 2011 Feb 2;6(2):e14610. doi: 10.1371/journal.pone.0014610 (PMC3032724; doi:10.1371/journal.pone.0014610)
Supplement: Table S1 — In the Colchester mandrill community, the Eye covering gesture is performed frequently by five members (one now deceased) and occasionally by two additional members. Information about the gesturers is provided below, along with each individual's respective sample size of gestures and other information about their gesturing. (0.04 MB DOC) [file pone.0014610.s001.doc]

**Table S1.** In the Colchester mandrill community, the Eye covering gesture is performed frequently by five members (one now deceased) and occasionally by two additional members. Information about the gesturers is provided below, along with each individual’s respective sample size of gestures and other information about their gesturing.

| ***Name*** | **Year gesturing first recorded** | **Total gestures**  **[No. in**  **focal sampling]** | **Percentage with**  **left, both, or right hand** | **Ever elbow raise?** | **Fine-scale bouts** **^A^**  Mean (sample size) and range | **Lumped bouts ^B^**  Mean (sample size) and range |
| --- | --- | --- | --- | --- | --- | --- |
| *Milly* | 1999 | N = 193  [n = 55 in focal] | 85.0, 0.5, 14.5 | Never | 32 s (n = 176)  Range: 1 s –  12.5 min | 10.0 min (n = 12)  Range: 2 s –  36.2 min |
| *Phoenix* | After 1999 **^C^** | N = 12  [n = 0 in focal] **^D^** | 41.7, 0.0, 58.3 | Y | NA | NA |
| *Mac* | After 1999 **^C^** | N = 53  [n = 15 in focal] | 25.0, 3.1, 71.9 | Y | 2.2 min (n = 46)  Range: 3 s – 17.1 min | 13.1 min (n = 9)  Range: 2.9 min –  28.4 min |
| *Max* | After 1999 **^C^** | N = 64  [n = 21 in focal] | 41.9, 16.3, 41.9 | Y | 1.0 min (n = 51)  Range: 2 s – 6.0 min | 4.7 min (n = 12)  Range: 3 s –  21.0 min |
| *Barney* | 2007 (or slightly before) | N = 28  [n = 2 in focal] | 66.7, 16.7, 16.7 | Y | 5.1 min (n = 2)  Range: 2.5 min – 7.7 min | 10.2 min (n = 1) |
| *T.J.* | 2007 | N = 4  [n = 0 in focal] **^E^** | 50.0, 25.0, 25.0 | Never | NA | NA |
| *Kayin* | 2008 | N = 5  [n = 0 in focal] **^F^** | 60.0, 20.0, 20.0 | Y | NA | NA |

**^A^** Represents the exact onset and offset of the gesture (i.e., from the time a specific hand was first placed over the eyes to the time it was taken off). The sample size is the number of separate onset-offset instances across all focal sampling of that individual.

**^B^** Represents the joining of adjacent bouts in which the gesturer switched hands or in which the gesturer took its hand off and then placed this same hand back on within 1 min. The separations of < 1 min in which the gesturer was no longer gesturing are included in the time; during these separations the gesturer either looked around, used its hand to scratch itself, changed positions slightly, or simply rested its arm.

**^C^** Phoenix, Mac, and Max began performing the gesture subsequent to Milly but before Barney; the exact year of each one’s first performance is not known.

**^D^** Phoenix died before focal observations could be carried out on him. He was injured by the alpha male (‘Dume’) before the second study period. After his wounds were stitched up twice (since he picked out the first set of stitches), it was decided he should be put to sleep; keeping him alive would have required use of an IV drip and total removal from the group.

**^E^** Focal samples were carried out on T.J., as he was ≥ 3 yrs in age and he was observed performing the gesture in the first study period. However, during none of the focal samples did he gesture, only during behavioral sampling.

**^F^** As Kayin was an immature and was never observed to perform the gesture in the first study period, no focal samples were carried out on him. However, near the end of the second study period he was observed performing the gesture in behavioral sampling.
