# Supplementary material for: Meaningful Gesture in Monkeys? Investigating whether Mandrills Create Social Culture
Source: PLoS One. 2011 Feb 2;6(2):e14610. doi: 10.1371/journal.pone.0014610 (PMC3032724; doi:10.1371/journal.pone.0014610)
Supplement: Table S2 — Parentage of Colchester mandrills that have performed and not performed the Eye covering gesture. (0.04 MB DOC) [file pone.0014610.s002.doc]

**Table S2.** Parentage of Colchester mandrills that have performed and not performed the Eye covering gesture.

| ***Name*** | **Mother** | **Father** |
| --- | --- | --- |
| GESTURERS |  |  |
| *Milly* **^A^** | Malaya | Male 74 **^C^** |
| *Phoenix* | Celine | Male 74 **^C^** |
| *Mac* | Celine | Male 2016 **^C^** |
| *Max* **^B^** | Orinoko | Male 2016 **^C^** |
| *Barney* **^B^** | Orinoko | Male 2016 **^C^** |
| *T.J.* | Solomina | Male 2016 **^C^** |
| *Kayin* | Celine | Dume |
|  |  |  |
| NON-GESTURERS |  |  |
| *Dume* | Another zoo – Southport | Another zoo – Southport |
| *Celine* | Another zoo – London | Another zoo – London |
| *Orinoko* **^A^** | Malaya | Male 74 **^C^** |
| *Oakley* **^B^** | Orinoko | Male 2016 **^C^** |
| *Malaya* | Another zoo – Paignton | Another zoo – Paignton |
| *Matilde* | Malaya | Male 2016 **^C^** |
| *Solomina* **^A^** | Malaya | Male 74 **^C^** |

**^A^** These three individuals comprise a set of full siblings.

**^B^** These three individuals comprise a different set of full siblings.

**^C^** These fathers were prior alpha males in Colchester (they preceded the current alpha male, Dume).
