# Supplementary material for: Meaningful Gesture in Monkeys? Investigating whether Mandrills Create Social Culture
Source: PLoS One. 2011 Feb 2;6(2):e14610. doi: 10.1371/journal.pone.0014610 (PMC3032724; doi:10.1371/journal.pone.0014610)
Supplement: Table S3 — Other study groups outside of Colchester [updated from Table 1 of Laidre 2008, 2009]. Groups are listed chronologically in the order in which they were first observed. Personal communications from other mandrill observers follow at the bottom of the Table. (0.06 MB DOC) [file pone.0014610.s003.doc]

**Table S3.** Other study groups outside of Colchester [updated from Table 1 of Laidre 2008, 2009]. Groups are listed chronologically in the order in which they were first observed. Personal communications from other mandrill observers follow at the bottom of the Table.

| Group ^A^ | Age-sex composition ^B^ | Enclosure ^C^ | Density ^D^ | Observation period (mm/yyyy) | Observation length (h) ^E^ |
| --- | --- | --- | --- | --- | --- |
| Syracuse | AM, AF, 2JF | Indoor (9 x 8) | 0.056 (7^th^) | 01/2002–05/2008 | 289-F, 6-B |
| Buffalo | AM ^F^, AF, 2JF | Outdoor naturalistic (12 x 6) | 0.056 (7^th^) | 08/2002–05/2008 | 114-F, 11-B |
| Tampa | 2AM, 2AF, 3JM, JF, IF | Outdoor naturalistic (14 x 6) | 0.107 (2^nd^) | 12/2002–01/2009 | 28-F, 22-B |
| Staten Island | AM, 3AF | Indoor (9 x 7) | 0.064 (5^th^) | 01/2003 | 50-F |
| Franceville 1 | 2AM, 10AF, 5JM, 3JF, 2IM, 3IF | Outdoor naturalistic (1.5 hectares) | 0.002 (18^th^) | 06/2004–09/2004 | >100-F, >100-B |
| Franceville 2 | 5AM, 10AF, 8SAM, 6SAF, 10JM, 12JF, 4IM, 7IF | Outdoor naturalistic (3 hectares) | 0.002 (18^th^) | 06/2004–09/2004 | >50-F, >50-B |
| Franceville 3 | AM, 2AF, JM | Outdoor (8 x 8) | 0.063 (5^th^) | 08/2004–09/2004 | 15-B |
| Paignton | AM, AF, IM, 2AF ^G^ | Indoor (8 x 6) & Outdoor naturalistic (15 x 15) | 0.032 (9^th^) | 03/2005 | 30-F, 5-B |
| San Francisco | AM, 3AF, JF | Outdoor naturalistic (14 x 14) | 0.026 (12^th^) | 10/2007 | 5-B |
| Portland | AM, 4AF | Indoor (10 x 4) & Outdoor naturalistic (15 x 8) | 0.083 (3^rd^) | 12/2007 | 6-B |
| Norfolk | 2AM, 2AF ^H^ | Outdoor naturalistic (30 x 10) | 0.007 (16^th^) | 02/2008–05/2010 | 4-F, 46-B |
| Boston | AF, SAM | Indoor (11 x 6) | 0.030 (10^th^) | 02/2009 | 6-B |
| Bronx | AM, 2AF | Indoor (10 x 14) | 0.021 (14^th^) | 04/2009–06/2009 | 45-B |
| Jacksonville | AF, JF | Outdoor naturalistic (30 x 20) | 0.003 (17^th^) | 04/2009 | 16-B |
| Miami | AM, 3SAM, AF, JF | Outdoor naturalistic (10 x 5) | 0.120 (1^st^) | 04/2009 | 7-B |
| Berlin | AM, 3AF, JF, JM, 2IM | Indoor (6 x 4) & Outdoor (40 x 25) | 0.008 (15^th^) | 04/2009-05/2009 | 15-B |
| Hamburg | 2AM, 7AF, 2SAM, SAF, 5JM, 5JF, 5IU | Indoor (7 x 9) & Outdoor naturalistic (310 m^2^) | 0.072 (4^th^) | 05/2009 | 11-F, 47-B |
| Rome | AM, 5AF, SAM, SAF, 3JM, 5JF, 2IF | Outdoor naturalistic (30 x 25) | 0.024 (13^th^) | 12/2009 | 25-B |

**^A^** Syracuse = Rosamond Gifford Zoo at Burnet Park (NY, USA), Buffalo = Buffalo Zoological Gardens (NY, USA), Tampa = Lowry Park Zoo (FL, USA), Staten Island = Staten Island Zoo (NY, USA), Franceville 1, 2, 3 = Centre International de Recherches Médicales (Gabon), Paignton = Paignton Zoo Environmental Park (UK), San Francisco = San Francisco Zoo (CA, USA), Portland = Oregon Zoo (OR, USA), Norfolk = Virginia Zoological Park (VA, USA), Boston = Franklin Park Zoo, aka Zoo New England (MA, USA), Bronx = Bronx Zoo, Wildlife Conservation Society (NY, USA), Jacksonville = Jacksonville Zoo and Gardens (FL, USA), Miami = Monkey Jungle (FL, USA), Berlin = Berlin Zoo (Germany), Hamburg = Tierpark Hagenbeck (Germany), Rome = Rome Zoo, aka Fondazione Bioparco di Roma (Italy)

**^B^** A = adult (older than 7 yrs), SA = subadult (5 - 7 yrs), J = juvenile (2 - 5 yrs), I = infant (< 2 yrs); M = male, F = female, U = unknown (in a few rare cases recently born individuals had not yet been sexed); Numerals preceding an age-sex abbreviation indicate the number of individuals of that age-sex class within the group

**^C^** Measures given as length x width (in meters), or as absolute area (1 hectare = 10,000 m^2^)

**^D^** Density given as number of individuals per m^2^, and in parentheses the groups are ranked from most to least dense; Colchester’s density (at its peak group size) was 0.027, or 11^th^ most dense of the 19 total study groups

**^E^** F = Focal animal sampling [Altmann 1974], B = Behavioral sampling [Martin and Bateson 1993, p. 87]

**^F^** Introduced to the group approximately halfway through the study

**^G^** These two adult females were housed in a separate sub-group

**^H^** The group was divided into two separate sub-groups, each with one AM and one AF

____________________________________________________________________________________________________________

**LITERATURE & PERSONAL COMMUNICATIONS.** In addition to the groups observed by the author, the behavior of a number of additional captive mandrill groups has been observed in fine detail by previous researchers whose publications are listed in the Introduction of Laidre [2008]. None of these publications mentions Eye covering, despite extensive overlap between the ethograms reported therein and the behaviors detected during my own mandrill observations. Supplementing this literature survey and my observations, I queried current mandrill researchers who have observed long-term in yet further communities:

*Stefano Vaglio* (email: [stefano.vaglio@unifi.it](mailto:stefano.vaglio@unifi.it); pers. comm. 16 Oct 08) has never observed Eye covering in any of the other groups he has studied in Rome (Italy) and Jerusalem (Israel) and, for shorter periods, in Ostrava (Czech Republic) and Antwerp (Belgium), totaling N = 58 mandrills. He has, moreover, seen the Colchester Eye covering culture first-hand, not just through pictures and videos.

*Katharine Abernethy* (email: [k.a.abernethy@stir.ac.uk](mailto:k.a.abernethy@stir.ac.uk); pers. comm. 3 Nov 09) and *Joanna Setchell* (email: [joanna.setchell@durham.ac.uk](mailto:joanna.setchell@durham.ac.uk); pers. comm. 16 Oct 08) have never seen Eye covering in any of the three semi-free ranging colonies held at the Centre International de Recherches Médicales in Franceville, Gabon. Two of these colonies (listed in the Table above as ‘Franceville 1 and 2’) I observed personally on a daily basis for several months, and one larger colony (that as of June 2004 had N = 77 mandrills) I saw during my study but never systematically observed. Altogether, these three colonies encompass hundreds of mandrills that have been studied across multiple generations for more than a decade (E. Jean Wickings, personal communication), with no mandrill ever observed covering its eyes.

*Sacha Engelhardt* (email: [sacha.engelhardt@videotron.ca](mailto:sacha.engelhardt@videotron.ca); Concordia University; pers. comm. 11 Nov 2010) and *Kléo Carrier* (email: [kleo.carrier.1@ulaval.ca](mailto:kleo.carrier.1@ulaval.ca); Université Laval; pers. comm. 12 Nov 2010) have never observed Eye covering in the 10 mandrills at the Granby Zoo in Montreal, Canada after 165 h and 80 h of independent non-overlapping observation, respectively.
