# Supplementary material for: Meaningful Gesture in Monkeys? Investigating whether Mandrills Create Social Culture
Source: PLoS One. 2011 Feb 2;6(2):e14610. doi: 10.1371/journal.pone.0014610 (PMC3032724; doi:10.1371/journal.pone.0014610)
Supplement: Table S4 — Rate (per h) of approaches received while individuals were gesturing and not gesturing. Times while individuals were not gesturing are divided into stationary periods (focal was sitting or lying) and moving periods (focal was locomoting about). (0.04 MB DOC) [file pone.0014610.s004.doc]

**Table S4.** Rate (per h) of approaches received while individuals were gesturing and not gesturing. Times while individuals were not gesturing are divided into stationary periods (focal was sitting or lying) and moving periods (focal was locomoting about).

|  | **Gesturing** |  | **_______________Not gesturing_______________** | | |  |
| --- | --- | --- | --- | --- | --- | --- |
|  |  | *Stationary*  *(sitting or lying)* | | *Moving* | *Stationary and Moving combined* | |
| Gesturers **^A^** |  |  | |  |  | |
| Milly | 9.05 | 14.93 | | 0.00 | 14.29 | |
| Mac | 4.00 | 18.60 | | 83.33 | 26.53 | |
| Max | 7.37 | 15.19 | | 54.84 | 26.48 | |
| Barney | 0.00 | 24.72 | | 78.21 | 40.89 | |
| T.J. | — **^B^** | 13.33 | | 45.10 | 22.04 | |
| ***Mean ± SE*** | ***5.10 ± 2.00***  (N = 4) | ***17.36 ± 2.03***  (N = 5) | | ***52.30 ± 14.88***  (N = 5) | ***26.05 ± 4.33***  (N = 5) | |
| Non-gesturers |  |  | |  |  | |
| Dume | NA | 32.68 | | 65.79 | 43.67 | |
| Celine | NA | 37.78 | | 30.40 | 33.33 | |
| Orinoko | NA | 24.56 | | 58.70 | 31.80 | |
| Oakley | NA | 3.61 | | 51.52 | 10.57 | |
| Malaya | NA | 15.06 | | 36.36 | 25.68 | |
| Matilde | NA | 14.38 | | 73.33 | 20.00 | |
| Solomina | NA | 16.81 | | 31.94 | 22.51 | |
| ***Mean ± SE*** | NA | ***20.70 ± 4.44***  (N = 7) | | ***49.72 ± 6.49***  (N = 7) | ***26.79 ± 4.03***  (N = 7) | |

**^A^** Phoenix and Kayin not included, given the absence of focal samples on them (see Materials and Methods and Table S1).

**^B^** T.J. never performed the gesture in any of the focal samples conducted on him; only during behavioral sampling did he gesture.
