# Supplementary material for: Meaningful Gesture in Monkeys? Investigating whether Mandrills Create Social Culture
Source: PLoS One. 2011 Feb 2;6(2):e14610. doi: 10.1371/journal.pone.0014610 (PMC3032724; doi:10.1371/journal.pone.0014610)
Supplement: Table S5 — Rate (per h) of touches received while individuals were gesturing and not gesturing. See Table S4 for further explanation. (0.04 MB DOC) [file pone.0014610.s005.doc]

**Table S5.** Rate (per h) of touches received while individuals were gesturing and not gesturing. See Table S4 for further explanation.

|  | **Gesturing** |  | **_______________Not gesturing_______________** | | |  |
| --- | --- | --- | --- | --- | --- | --- |
|  |  | *Stationary*  *(sitting or lying)* | | *Moving* | *Stationary and Moving combined* | |
| Gesturers **^A^** |  |  | |  |  | |
| Milly | 0.50 | 1.49 | | 0.00 | 1.43 | |
| Mac | 0.00 | 2.33 | | 10.61 | 3.34 | |
| Max | 1.05 | 0.63 | | 1.61 | 0.91 | |
| Barney | 0.00 | 2.50 | | 12.82 | 5.62 | |
| T.J. | — **^B^** | 0.74 | | 1.96 | 1.08 | |
| ***Mean ± SE*** | ***0.39 ± 0.25***  (N = 4) | ***1.54 ± 0.39***  (N = 5) | | ***5.40 ± 2.62***  (N = 5) | ***2.48 ± 0.90***  (N = 5) | |
| Non-gesturers |  |  | |  |  | |
| Dume | NA | 0.00 | | 1.32 | 0.44 | |
| Celine | NA | 6.67 | | 4.00 | 5.09 | |
| Orinoko | NA | 2.92 | | 15.22 | 5.53 | |
| Oakley | NA | 0.00 | | 0.00 | 0.00 | |
| Malaya | NA | 1.20 | | 3.03 | 2.11 | |
| Matilde | NA | 5.48 | | 13.33 | 6.25 | |
| Solomina | NA | 0.84 | | 1.39 | 1.05 | |
| ***Mean ± SE*** | NA | ***2.45 ± 1.02***  (N = 7) | | ***5.47 ± 2.33***  (N = 7) | ***2.92 ± 0.99***  (N = 7) | |

**^A^** Phoenix and Kayin not included, given the absence of focal samples on them (see Materials and Methods and Table S1).

**^B^** T.J. never performed the gesture in any of the focal samples conducted on him; only during behavioral sampling did he gesture.
